# Clinical Review

**Published:** 1839

**Authors:** 


### Clinical Review.

---

### GUY'S HOSPITAL.

Guy's Hospital Reports. No. VIII. April, 1839. Edited by George H. Barlow, M.A., &c. and James P. Babington, M.A. &c.

The present number offers the following physiological and pathological *carte* :—

1. On the Disorders of the Brain, connected with Diseased Kidneys; by Thomas Addison, M.D.

2. On Perforations of the Stomach, from Poisoning and Disease; by Alfred S. Taylor. (With Plate).

3. On the Diurnal Variations of the Pulse; by William Augustus Guy, M.B. Cantab.

4. Observations on Poisoning by the Vapours of Burning Charcoal and Coals; by Golding Bird, M.D. F.L.S.

5. Two Cases of Poisoning by the Inhalation of Carburetted Hydrogen; by Thomas Pridgin Teale, F.L.S.

6. Case of Imperforate Uterus, with Remarks; by Alexander Tweedie.

7. On Incision in Cases of Occlusion, and Rigidity of the Uterus; by Samuel Ashwell, M.D.

8. Observations on Fibrinous Concretions in the Heart; by Dr. Hughes, M.D. F.L.S. (With Plates).

9. Analysis of Bones affected with Mollities Ossium; by G. O. Rees, M.D. F.G.S.

10. Case of Division of the Tibia, for the cure of Deformity occasioned by a Gun-shot Wound; by Charles Aston Key. (With Plate).

11. Case of Spermatocele, or Varicocele, treated by excision of a portion of the Scrotum; by Bransby B. Cooper, F.R.S.

12. Observations on Abdominal Tumors and Intumescence; illustrated by Cases of Renal Disease. By R. Bright, M.D. F.R.S. (With Plates.)

The first paper to which we shall allude is that by Dr. Addison, which, indeed, ushers in the volume.

### I. On the Disorders of the Brain connected with Diseased Kidneys. By Thomas Addison, M.D.

This able and observant Physician has directed his attention to a point of pathology or rather of semeiology, which is nearly, if not altogether, new ground. The object is threefold—

"*First*, To point out the general character and individual forms of cerebral disorder connected with interrupted function of the kidneys, from whatever cause such interrupted function may arise. *Secondly*, To shew, that, in recent as well as in chronic disease of the kidney, the cerebral disorder is not unfrequently the most prominent, and occasionally the only obvious symptom present. And *Thirdly*, To establish a means of diagnosis, in such obscure or in unsuspected cases, upon the peculiar character of the cerebral affection."

The reciprocal action of the brain on the kidney, and the kidney on the brain, has long been known; but we are not aware that any attempt has been made to specify with precision, and in detail, the several forms of cerebral disorder connected with, or dependent on renal affection—and to ground a diagnosis of the *latter* on the character of the former—that is to say, to tell whether disorder of the kidney exists, when only the cerebrum offers morbid phenomena, and when there is no symptom of nephritis, dropsical effusion, or albuminous urine. We cannot abbreviate the following passage without detriment.

"According to my experience, the general character of cerebral affections connected with renal disease is marked by *a pale face, a quiet pulse, a contracted or undilated and obedient pupil, and the absence of paralysis*:—this general character, however, being somewhat modified, in certain cases, by circumstances attending the individual attack.

So far as I have yet been able to observe, the individual forms of cerebral disorder connected with renal disease are the five following:—

1. A more or less sudden attack of *quiet stupor;* which may be temporary and repeated; or permanent, ending in death.

2. A sudden attack of a *peculiar modification of coma and stertor;* which may be temporary or end in death.

3. A sudden attack of *convulsions;* which may be temporary or terminate in death.

4. *A combination of the two latter;* consisting of a sudden attack of coma and stertor, accompanied by constant or intermitting convulsions.

5. A state of *dulness of intellect, sluggishness of manner,* and *drowsiness,* often preceded by *giddiness, dimness of sight,* and *pain in the head;* proceeding either to *coma* alone, or to *coma accompanied by convulsions;* the coma presenting the peculiar character already alluded to.

With respect to the first-mentioned form of cerebral disorder connected with renal disease, that of quiet stupor, it is, in its most exquisite form, probably the least frequently met with; the face is pale, the pulse quiet, the pupil natural, or at least obedient to light; and although the patient may lie almost completely motionless, there is no paralysis; for, on attentively watching him for some time, he will be observed slightly to move all the extremities. By agitating him and speaking loudly, he may sometimes be partially roused for a moment, but quickly relapses into stupor, as before; or it may not be possible to rouse him at all. There is little or no labour of respiration, no stertor, and no convulsions. Slight degrees of it occasionally precede and pass into the next or second form.

This second form of cerebral affection is that of a sudden attack of coma with stertor, or, in other words, apoplexy: it is, nevertheless, different from ordinary apoplexy: it is the serous apoplexy of authors, and presents the usual general characters of cerebral affection depending upon renal disease; for the face, instead of being flushed, is, in almost every instance, remarkably pale; the pulse, though sometimes small, and more rarely full, is remarkably quiet, or almost natural; the pupil, also, although occasionally dilated or contracted, is often remarkably natural in size, and obedient to light; and there is no paralysis. When the labour of respiration is very great, the general character is apt to be modified by an accelerated pulse, and occasionally by a slight flush of the countenance. The coma is for the most part complete, so that the patient cannot be roused to intelligence for a single moment. The stertor is very peculiar, and in a great measure characteristic of this form of cerebral affection connected with renal disease: it has not by any means, in general, the deep rough, guttural, or nasal sound of ordinary apoplexy: it is sometimes slightly of this kind; but much more commonly the stertor presents more of a hissing character, as if produced by the air, both in inspiration and in exspiration, striking against the hard palate or even against the lips of the patient, rather than against the velum and throat, as in ordinary apoplectic stertor: the act of respiration, too, is usually, from the first, much more hurried than is observed in the coma of ordinary apoplexy. The peculiar stertor coupled with the pale face has, in more instances than one, enabled me to pronounce with confidence the disease to be renal, without asking a single question, and in cases, too, in which no renal disease whatever had for a moment been suspected.

The third form of cerebral disorder connected with renal disease is that of a sudden attack of convulsions. In this case, also, the countenance is, for the most part, remarkably pale, although occasionally, slightly flushed at intervals: the pupil is often but little affected: in slight attacks of the kind, the pulse is sometimes singularly quiet; but when the convulsions are severe, and especially when there is such a degree of coma as to be attended with stertor, the heart often sympathizes, and the pulse becomes rapid, irregular, and jerking. This form of cerebral affection often passes into the fourth variety; or the cerebral affection shall take on the form of the fourth variety from the commencement: in the latter case we have merely a combination of the second and third varieties —the coma, hurried breathing, stertor, and convulsions being so blended together as often to have led to a dispute, whether the affection ought to be designated apoplexy or epilepsy. From what has been already stated, it may, in general be very easily recognised as one of the common forms of cerebral disorder, connected with renal disease.

The fifth variety is that in which the cerebral disorder makes its approach in a more gradual and insidious manner, usually commencing with dulness of intellect, sluggishness of manner, and drowsiness, gradually proceeding to coma, and more or less stertor, with or without convulsions; these states being, at the same time, distinguished by the general indications already pointed out. This form of cerebral disorder appears to be that which most commonly supervenes in the progress of the morbid change of kidney described by Dr. Bright; and is very frequently preceded by giddiness, dimness of sight, and pain in the head."

A highly interesting question is—whether there really exists any discoverable relation between the character of the renal and the cerebral affection? And if there be, in what relation are the forms, violence, and permanence of the latter to the former? Our author acknowledges that he is not yet in possession of sufficient facts to justify any very positive conclusion on these points, although he imagines that he has perceived a certain degree of relation between the affections of the two organs.

"Of all the more serious affections of the brain arising in connection with renal disease, the mildest form appears to be that of a tendency to a state of quiet stupor, varying in degree from a mere torpidity of manner and sluggishness of intellect, to complete insensibility to all surrounding objects. Accordingly, I have found this form of cerebral disorder most frequently present in what may be regarded as the least formidable, or more temporary derangements of the kidney. The most exquisite example I ever saw, occurred in a man who at the time presented no dropsical symptom whatever, whose urine was not albuminous, and who made no complaint of pain or uneasiness in his loins. After death, the cortical part of the kidneys was found highly injected, of a deep-red or almost chocolate colour, and somewhat softened in its texture; in short, furnishing the strongest indications of a recent nephritic attack in a subdued form: it is also my belief, that the same state of things not unfrequently takes place, at an early period in the progress of scarlatina: we observe an approach to a similar condition of brain in cases of fever, in which the bladder has been allowed to become over-distended; and most assuredly in cases of retention from stricture, and in cases of calculus in the kidney. In all these instances, the interruption or impediment to the urinary secretion may be said to be recent or incomplete; and hence, probably the less degree of severity of the cerebral affection, and the less peril to the patient; for in such instances the symptoms very commonly pass away, and the patient recovers. When, however, the hurtful cause is of an originally nephritic character, the chance of recovery will be less than when the cause of obstruction happens to be merely mechanical and temporary.

The next, in point of severity, of the cerebral affections connected with renal disease appears to be that of convulsions, with comparatively little stertor;—convulsions, however, which may prove speedily fatal; or which may be repeated an indefinite number of times, but from which the patient very often completely and permanently recovers. Accordingly, I have observed this form of more simple convulsions most frequently associated with what may fairly be regarded as a more exquisite and enduring form of renal disease than that just alluded to: I have observed it most frequently in cases of renal dropsy, subsequent to scarlatina; and in that form of renal dropsy supposed to arise from direct exposure to damp and cold, and commonly known by the name of inflammatory dropsy. As the renal affection has already proceeded to induce dropsy, we cannot but regard it as more fixed and more formidable than in the cases described as being attended with more or less of quiet stupor: and accordingly, instead of merely a certain degree of this latter condition, we have convulsion which may indeed prove fatal, but from which, as already observed, the patient often completely and permanently recovers."

The most stubborn and dangerous cases of cerebro-renal affection are those where the structure of the kidney is irrecoverably disorganized in the manner described by Dr. Bright. It is not true, however, that every such case is associated with cerebral disorder—on the contrary, many cases occur where the sensorial functions remain unaffected till the very last period of the renal complaint. What it is which causes this discrepancy, we are, at present, unable to ascertain. It has appeared to Dr. Addison, that when the cerebral affection does supervene on this fatal form of renal disease, its constancy, urgency, and intractability correspond with that of the primary malady.

"The patient suffering repeatedly, or more or less constantly, from heaviness, drowsiness, giddiness, or pain or sense of tightness in the head, and being peculiarly liable to be suddenly seized with the most alarming and most fatal of all the forms of cerebral disorder occurring in connection with renal disease—profound coma and stertor, with or without convulsions."

The post-mortem appearances in the brain are passed over by our author—as they are very often slight, and apparently inadequate to an explanation of the symptoms.

We are much obliged to Dr. A. for breaking ground in an untrodden path of inquiry, and have no doubt that he will prosecute the investigation with that zeal and talent for which he is distinguished.

### II. ON THE DIURNAL VARIATIONS OF THE PULSE; BY WILLIAM AUGUSTUS GUY.

We have already noticed some observations of Dr. Guy's upon the pulse. Those before us now are intended to combat the popular notion, that the pulse is more frequent in the evening than in the morning, a notion which was first opposed by Dr. Knox.*

The observations of Dr. Guy are too voluminous to permit us to present them in detail. We shall therefore content ourselves with chronicling the main results.

The experiments were made at Cambridge, in the Spring of 1837. Dr. Guy rarely went to bed before midnight, often as late as one or two o'clock in the morning; he usually rose at nine o'clock.

* "On the relation subsisting between the time of the day and various functions of the human body, and on the manner in which the pulsations of the heart and arteries are affected by muscular exertion." By Robert Knox, M.D. Edinb. Ed. Med. and Surg. Journal, Vol. XI. p. 53.

Twenty observations made on first rising in the morning—and twenty at night, just before going to bed, and after remaining quiet for some hours—gave the following numbers; the sitting posture being maintained in all the observations.

| | Morning. | | Night. | | Difference. |
|---|---|---|---|---|---|
| Max. ........ | 68 | .. | 61 | .. | 7 |
| Min. ......... | 54 | .. | 50 | .. | 4 |
| Mean. ...... | 64 | .. | 54 | .. | 10 |

Here, then, the pulse was more frequent in the morning than at night, by 10 beats; and there was a difference, between the highest frequency in the morning and the lowest frequency at night, of 18 beats. This remarkable diminution towards night took place in spite of the various excitements produced by food, study, or exercise during a space of fifteen or sixteen hours.

2. The following remarks on the effects produced by rest upon the pulse are not undeserving of attention.

"Though the medical man is familiar with the rapid diminution of frequency which the pulse undergoes after being raised above its natural number by food, exercise, or mental excitement, the effect of long-continued rest, in diminishing what may be termed its normal frequency, has not attracted much attention even from those who have most carefully investigated the diurnal revolutions of the pulse. If we take the frequency of the pulse on first rising in the morning, and before it has been subject to the excitement of food or exercise, as a standard of comparison, we shall find that continued rest will greatly reduce that frequency. If, again, after the pulse has been increased in frequency by food, we allow it to subside to the number which it had before the food was taken, and then continue for a considerable period in a state of rest, the pulse becomes less and less frequent, till it reaches a much lower frequency than that which it had on first rising in the morning. My first series of observations illustrates this position. But this effect takes place at all periods of the day, in the morning as well as in the evening. Are we justified, then, in regarding every progressive decrease in the frequency of the pulse as evidence that the pulse becomes less frequent as the day advances?—Certainly not. The only way, therefore, in which we can determine whether the pulse is really less frequent at night than in the morning, is by comparing the morning with the evening pulse under precisely the same circumstances. That I might make this comparison, I adopted a plan somewhat similar to the one suggested by Knox. I rose between eight and nine A.M.; and, when dressed, remained in the sitting posture, and in a state of perfect rest, for some minutes. I then counted the pulse; and took its frequency at that period as my standard of comparison throughout the day. I then ate my breakfast, which consisted of three cups of tea, and the half of a small loaf of bread and butter. Immediately after the meal, I again counted the pulse; and repeated my observations every quarter of an hour, until it had reached the same frequency which it had before the meal. From this time I continued to count the pulse every quarter of an hour, during a period varying from half-an-hour to two hours. From three to four o'clock I took a walk; and dined at four. After dinner I remained at rest till my pulse acquired the frequency which it had on first rising in the morning and before breakfast. At this point of time, which was generally between eight and nine o'clock, I took my tea, consisting of the same food, and in precisely the same quantity, as that which had formed my breakfast. After tea, I counted my pulse every quarter of an hour, till it fell to the same frequency which it had before the meal. I then repeated the observations of the morning, counting the pulse every quarter of an hour till twelve, one, or two o'clock; and taking care to make precisely the same number of observations as I had already made in the morning. I thus obtained two series of observations, in every respect analogous, and admitting of the most exact comparison. That no error might creep in, I not only remained during the whole time of the observations at rest and in the same posture, but I took care to pursue precisely the same study in the morning as in the evening."

Omitting the table of results we will insert the summary of them.

*a.* It appears that the pulse is less frequent late at night than at nine A.M. by from four to six beats;* and that during the same interval of time it falls more rapidly, and attains a lower frequency in the evening than in the morning. The difference, indeed, is in no case very considerable, but it is remarkable for the uniformity of its occurrence; and it is well worthy of remark, that the same difference which occurs in the mean of all the observations takes place in each separate series; a circumstance which, as it is rarely met with in experiments on the human body, will inspire the greater confidence in the results obtained. In five out of the eleven series of observations, irregularities were observed in the morning; that is to say, the diminution of the pulse was not progressive, but an occasional increase of frequency took place. In the evening, the diminution was, in every case, uniformly progressive.

*b.* Out of fourteen observations, there is one only in which the immediate effect of the meal was greater in the evening than in the morning, and then only by one beat; and one only in which the effect was equal at both times in the day: whilst in every case, without exception, the effect was of shorter duration in the evening than in the morning. But by far the most remarkable fact established by these observations, is, that the same food which in the morning increases the frequency of the pulse from five to twelve beats, and keeps it raised above its natural number during from one to two hours, may in the evening produce no effect whatever.

3. Dr. Guy sums up thus:

1. The pulse of a healthy adult male in a state of rest, unexcited either by food or exercise, is most frequent in the morning, and gradually diminishes as the day advances.

2. The pulse diminishes in frequency more rapidly in the evening than in the morning.

3. The diminution of the frequency of the pulse is more regular and progressive in the evening than in the morning.

4. The effect of food is greater and more lasting in the morning than in the evening; and, in some instances, the same food which in the morning produces an effect considerable both in amount and in duration, has no effect whatever in the evening.

Dr. Guy is evidently possessed of a clear head and of professional zeal. Those useful qualities lead us to expect, and will enable him to present, contributions to practical medicine of greater value than those which he has yet offered.

### III. ANALYSIS OF BONES AFFECTED WITH MOLLITIES OSSIUM. By G. O. REES, M.D. F.G.S. &c.

In a Paper published in the 21st Volume of the Medico-Chirurgical Transactions, and noticed by us at the time, Dr. Rees, an able and indefatigable chemist, demonstrated by analysis, that the different bones of the adult human

---

* "Immediately on getting out of bed, the pulse is more frequent than after an interval; so that the mean difference between the pulse at that time and late at night is, as we have already seen, about ten beats."

skeleton, in health, contained animal and earthy matter in different proportions. He has lately had an opportunity of examining some bones affected with mollities, and the following is the result of careful analyses of three specimens from the same adult subject; they are compared with those obtained from healthy bones.

| | MOLLITIES. | | | HEALTH. | | |
|---|---|---|---|---|---|---|
| | Earths. | | Animal Matter. | Earths. | | Animal Matter. |
| Fibula...... | 32.50 | .. | 67.50 | 60.02 | .. | 39.98 |
| Rib......... | 30.00 | .. | 70.00 | 57.49 | .. | 42.51 |
| Vertebra.... | 26.13 | .. | 73.87 | 57.42 | .. | 42.58 |

Dr. Rees observes :—

"On examining this Table, it will be observed that in the diseased as well as in the healthy bones, the fibula contains more earthy matter than either the rib or vertebra; and the rib more than the vertebra :—thus we have the same order preserved as in health. It may be noticed that the vertebra and rib, in health, approach very nearly in their proportions of animal and earthy matter; while in mollities, a considerable difference exists between them in this respect. This indicates, that though the bones are all acted upon by the absorbents in mollities, yet that the absorption does not go on equally in the bones; some being acted upon more than others. There is, however, an approach to an equality of action: for, notwithstanding that the diseased bones have lost about half of their earthy matter, yet they keep the same order, as regards proportional constitution, which we observe in health; viz. the fibula containing more earthy matter than the rib, and the rib more than the vertebra.

Having ascertained, by previous experiment, that the earthy matter obtained from the long bones of the extremities contained, as nearly as possible, 86 per cent. of phosphate of lime in health, and that the earthy matter from the trunk bones contained, on an average, *83.03 per cent.; I determined on mixing together the earths obtained from the fibula, rib, and vertebra affected with mollities, and subjecting them to analysis. This bone-earth, on examination, proved to contain only 78 per cent. of phosphate of lime. There is evidence here, that the absorption of earthy matter by disease is accompanied by a decrease in the proportion of phosphate of lime to carbonate. This would seem to shew that the absorbents carry away earthy matter containing a very large per centage of phosphate of lime: for were it otherwise, we should never find bone-earth containing so small a per centage as 78 of that earth; the smallest, in health, being 81.2 of phosphate, to 18.8 of carbonate of lime.

That the carbonate is absorbed together with the phosphate of lime, in certain though small proportion, in these processes, is proved by the fact, that bones, even those most changed by the absorption of their earthy matter, are not very greatly removed from healthy bone-earth in the general average proportion of phosphate and carbonate of lime."

### IV. Case of Spermatocele, or Varicocele, treated by Excision of a Portion of the Scrotum. By Bransby B. Cooper, F.R.S.

Our readers must be aware that Sir A. Cooper has proposed excision of a portion of the scrotum as a remedy for varicocele. Mr. Bransby Cooper publishes

* This average was drawn from results obtained from seven specimens: the extremes were 81.2 and 85.0.

the following case as a highly satisfactory instance of the benefit that may accrue from the performance of the operation.

*Case.* About three months ago Mr. Cooper was consulted by a young farmer, who had laboured under varicocele on the left side for upwards of two years. Mr. C. found the scrotum nearly double its natural size; and on the left side so pendulous, as to reach at least a third lower than on the opposite side. The slightest manipulation produced considerable pain, both in the testicle and in the course of the cord; and the testicle itself was wasted, and exhibited all the usual signs of an irritable condition.

"The patient complained of a constant sense of weight, attended with a heavy dull pain, extending from the testicle, along the spermatic-cord, to the loins, with an habitual feeling of restlessness and anxiety. His appetite was impaired, and a constant depression of spirits induced him to seek medical relief. All the usual remedies had been employed, as purging, recumbent posture, suspensory-bandages, and cold applications; but ineffectually. On questioning the patient as to the probable cause of the complaint, he could attribute it to none of the usual local causes, as a blow, &c. but admitted that he had always been more or less affected with a constipated state of bowels. I therefore proposed to him, that he should return into the country, submit himself to the continued influence of purgative medicine for a short time, abstaining from all violent exercise; and that if this plan did not remove his disease, I would attempt a radical cure, by the operation of excision of a portion of his scrotum. To this he readily acceded; seeming willing to submit to any temporary pain, rather than bear his protracted suffering. He therefore returned home, strictly adhered to the prescribed discipline for six weeks: at the end of which period, finding himself in no way improved, he came to London, determined to undergo any operation."

On the 8th of February, Mr. C. excised a portion of the scrotum in the following manner:

"The patient being placed in the recumbent posture at the foot of the bed, the enlarged veins of the left spermatic-cord were emptied of their blood; when Mr. Birkett drew the relaxed skin of the scrotum tightly between his fore and middle finger, so as to press the testicle closely against the external ring with the back of his hand. I then, with one sweep of the knife, removed the whole of the skin restricted by Mr. Birkett's finger, taking care to avoid the septum scroti: and thus exposed the tunica vaginalis, from which alone the left testicle now received any covering. The bleeding being very inconsiderable, I immediately proceeded to bring the edges of the incised skin together, by raising the lower portion towards the upper; and maintained the coaptation by three or four sutures; by which means I diminished the size of the left side of the scrotum, so as to form a close envelop to the testicle. The parts were then supported by adhesive-plaister and bandages, pretty tightly applied; and the whole was enveloped in cloths kept constantly moist with cold water."

Some degree of inflammation of the spermatic cords, and of the right testicle followed, suppuration occurred in the wound, but on the 4th of March he was convalescent. The spermatic cords, though firmer, were not larger than natural, and all appearance of varicocele had ceased.

Mr. Cooper remarks:—

"Various modes of treating varicose veins have been recommended—excision, division, application of ligatures, caustic and pressure, all with a view of obliterating the diseased vessel: but these means so frequently lead to phlebitis, and the consequent violent constitutional derangement, as to have led surgeons rather to adopt palliative means, than to grapple at once with the dangers inseparable from the attempts at a radical cure. The usual means employed to relieve spermatocele are, suspensory-bandages, for the purpose of diminishing the length, and consequently the weight of the column of blood contained in the veins—the application of evaporating lotions, to produce the constricting influence of cold—and the administration of purgatives, to prevent accumulation of the contents of the large intestines. Nothing further, however, can be expected from this plan than relief from the urgency of symptoms; unless, as sometimes happens during this treatment, the veins become obliterated by the spontaneous deposition of coagula within them. Sir A. Cooper, considering that the constant use of the suspensory-bandage, and application of evaporating lotions, were highly inconvenient, if not in themselves pernicious, believed that, by the excision of a large portion of the skin of the scrotum, he should at once produce all the desired results of the suspensory-bandage, without the necessity for its permanent application, and, at any rate, would get rid of its inconvenience: it was with the view, therefore, of thus establishing a continued well-adjusted bandage that he recommended the operation.

It appears to me, however, and indeed seems apparent, from the daily report of the above-related case, that the excision of the portion of the scrotum leads to the cure of spermatocele, by inducing inflammation, and consequent obliteration of the diseased veins; and without the same risk as attends upon the application of any immediate means to the veins themselves, as must be the case either by the employment of a ligature or the excision of the varix.

The history of the case plainly indicates the progressive symptoms, from the first inflammation of the spermatic-veins, to the period of their ultimate obliteration."

We would observe that the operation of excision of a portion of the scrotum was founded on the idea, that, by its cicatrization and contraction, it would prove a sort of bag-truss. We have not seen the opeiation followed by inflammation and obliteration of the veins. If, in Mr. Cooper's case it was so, that would obviously render it more effectual. But it seems difficult to suppose that it would not render it, pari passu, more unsafe. Phlebitis is always dangerous, whatever may have been its exciting cause. We have seen a patient die of inflammation of a varicose saphena vein, induced by merely tapping it with the fingers smartly through the skin. Considerations of this nature, coupled with the actual occurrence of phlebitis in the present instance, would lead us to suspect that even excision of a bit of the scrotum *may* be followed by serious results. At the same time it carries with it fewer disadvantages than any other efficient operation for varicocele.

### V. Case of Division of the Tibia, for the Cure of Deformity occasioned by a Gun-shot Wound. By Charles Aston Key.

Captain Charlton was wounded on the 17th of August, 1835, in capturing a stockade in Assam; a musket-ball fracturing the right tibia. His situation precluded proper assistance, and in March, 1838, he embarked for England.

"When he arrived in England, he walked with difficulty, supporting himself with two sticks, and carrying the broken limb at a considerable distance from the other, in order to bring the leg into a more perpendicular line of bearing. The deformity of the limb was great, to the full extent represented in the drawing. It appeared from the history of the accident, and subsequent treatment, that a considerable portion of bone having been lost on the inner side of the tibia, the broken ends had united at an angle, in the same manner, and from the same cause as an ulcerated spine acquires an irregular form from loss of substance on the anterior part of the vertebræ. The upper part of the tibia had not only formed an angle at its point of union with the lower portion of the bone, but also deviated from its natural line in relation to the femur. Its head, with the articulatory surface, had been somewhat forced outwards, so that an appearance of obliquity was given to it when viewed from before. In addition to the great deformity of the tibia, the fibula had undergone a displacement at its upper extremity. Its head had been forced away from its articulation with the tibia, and formed an unnatural prominence, above the usual position, in reference to the tibia. The bearing of this bone was also altered, as appears in the drawing. Not having been broken at the time of the accident, it could not yield and form an angle, as the tibia had at the seat of fracture; but maintaining its natural straight line, it had been compelled to alter its line of bearing, in compliance with the angular form of the larger bone. The fibula, therefore, preserved a line parallel to the lower portion of the tibia. Its lower end, being forcibly acted on by the inward inclination of the foot and lower part of the tibia, had carried the upper part outward; and had caused a dislocation of its head, which had undergone some change of form, and possessed a degree of motion not natural to it in its ordinary position. The shortening of the whole limb occasioned by this alteration in form was such as to cause Captain Charlton to walk on his toes; the heel being raised an inch and a half, when he stood upright. The soft parts had a healthy aspect; and the cicatrix over the bone had not contracted a firmer union to the periosteum than is usual with wounds situated directly over bone."

This description might, perhaps, be simplified by stating, that the tibia, near the junction of its upper and its middle thirds, formed an angle of between 130° and 140°, salient outwards.

On the 10th of October, Sir A. Cooper made, with Mr. Key, a careful examination of the limb: and after hearing the history of the accident, and its consequences, he decided that the limb might be restored to a useful state, and that the deformity might be remedied. He suggested, as the only means of restoration, that the bones of the leg should be divided—doubting whether the division of the tibia alone would be sufficient to set the fibula at liberty;—but that the tibia should first be divided; and, if necessary, the operation should be performed on the fibula. This was carried into effect on the 14th.

"At the operation were present Sir Astley Cooper, Mr. Atkins, surgeon, who accompanied Captain Charlton from India, and under whose advice he acted, Mr. Balderson, and my pupil, Mr. Montefiore. The tibia was laid bare on its anterior surface, by a longitudinal incision nearly four inches long, which traversed the line of the old wound, and allowed the integuments to be detached on each side; so that the anterior spine and the attachment of the soleus were exposed, just above the site of the old fracture. A strong steel grooved director, slightly curved, such as I use in operating for hernia, but narrow, was then passed along the outer surface of the tibia, detaching the tibialis anticus, until it reached the unyielding interosseous ligament. By a little firmer pressure, the director pierced it close to the bone. With another similar director, and by the same process, the inner and back surface of the tibia was so far detached from its muscles, that the ends of the two directors met behind the bone.

A curved needle, on which was hooked a chain-saw, was then passed along the groove of the outer director, and from thence to the groove of the inner; and its point being brought to view by a pair of dressing-forceps, the saw was adjusted so as to cut the bone from behind. When the tibia was about half sawn through, the saw—as chain-saws too often do, even when lightly used—locked, and became useless: the section of the bone was therefore completed from before, by a small common saw.

As soon as the tibia was divided, Sir Astley Cooper, taking the foot in his hand, found the lower part of the leg quite free to move in any direction, and that it was unnecessary to divide the fibula. As soon as the tibia was brought into a straight line, the head of the fibula was restored in some measure to its natural position, and ceased to project in the unseemly manner it had done before the operation. The part where the tibia was divided gaped, as soon as the bones were straightened; and the point of contact between the sawn ends of the tibia was but a small portion of its outer circumference. The muscles had, from length of time, acquired so fixed a state, that some force was required to overcome their resistance: for as soon as the hand was removed from the foot, they immediately carried it inwards, to its former position."

The limb was allowed to remain unconfined on pillows, until granulation was established. At the end of about ten days the wound had quietly gone through its several stages, and had healed, with the exception of about an inch in the centre: this part continued more or less open for some weeks, discharging a healthy pus, and giving exit to a few minute portions of exfoliating bone.

The limb, throughout the whole treatment, was kept upon the heel, with the knee straight. At first, two long lateral splints, well padded, were applied, so as to embrace the foot on each side: to these were added, afterwards, an under splint, to give more effective support and steadiness to the limb. The constant tendency to displacement was not effectually prevented by common tapes and bandages: as these became slack, the leg assumed its former distorted position; and thus motion was given to the broken ends of the bone, in adjusting the line of the limb. To avoid this, which was not practicable by means of straps or bandages, a tourniquet was applied at either end of the splint. The length of lever enabled the upper tourniquet to act with great power on the foot, and to keep it in a straight line with the thigh. The lower one was kept firmly screwed to the heel of the two splints; thus keeping the foot firmly secured, and giving steadiness to the action of the upper tourniquet. This plan of keeping the tapes of the tourniquets tight, prevented motion or displacement in the ends of the bones. It was at one time contemplated to substitute the white-of-egg bandage, in place of the splints; but the attempt was unsatisfactory, as displacement of the limb gradually took place.

Firm union was not obtained before the beginning of January. Even then the fibula had not become quite stable in its new position, and splints and common bandages were deemed requisite. The length of the limb appeared to be little curtailed.

The patient soon left town under the care of Mr. Atkins, and, on the 10th of March, that gentleman informed Mr. Key, by letter, that the long splints had been discontinued, and that the leg maintained as good a position as when he left town. Several small portions of bone had come away, which had retarded the cicatrization of the wound. In other respects he was proceeding well.

### VI. Case of Imperforate Uterus, with Remarks. By Alexander Tweedie.

The fourth number of Guy's Hospital Reports contained the history of a case of pregnancy with imperforate uterus. The subject of that case has again come under Mr. Tweedie's care.

Mrs. P. aged between 25 and 26, called December 31, 1838, to request Mr. Tweedie to attend her in her approaching confinement. Early in the morning of the 2nd of January, he was summoned to her. She had experienced slight pains on the 31st. They increased, and about the middle of the night of the first of January, the waters broke suddenly as she turned in bed.

Being much engaged, Mr. Tweedie sent an intelligent pupil, Mr. Batchelor to see the patient. He returned at noon, and reported she was indeed in labour, and the pains were of the most powerful kind: there was an opening into the uterus, through which he could feel the head presenting; but it had not dilated in the least during the time of his stay in the house; and altogether the state of parts was different from any thing he had before felt: he had given her half a drachm of laudanum, before leaving her. About two P.M. Mr. Tweedie saw her. The uterine contractions were intensely powerful and constant, resembling those produced by the full action of ergot of rye.

"On examination, the pelvis was found amply capacious. At the uterine extremity of the vagina (which is short, and she is a little woman) was an irregular opening, which, posteriorly, and laterally, seemed continuous almost with the vagina, but anteriorly, was bounded by a strong, firm, unyielding rigid edge, upon which, at each pain, the child's head was forcibly impelled. The entire opening might be about the area of a penny, rather less than more; and upon the anterior edge was plainly felt a cicatrix of the original incision, passing forwards towards the left ilio-pubic junction. There was no trace of cervix uteri. I left her under Mr. Batcheler's care; partly to try the efforts of nature somewhat longer, but principally to seek the advice of Dr. Ashwell. The doctor was not at home: so I returned about six o'clock P.M. in company with my friend and colleague, Mr. Lever.

We found our patient still suffering from undiminished pain; and the opening had not perceptibly enlarged since Mr. Batcheler had first seen her in the morning. The pulse was quickening, the skin was hot, the vagina was becoming hot and dry. We deemed it advisable to wait no longer: therefore, having emptied the bladder, I introduced two fingers of the left hand as far as the orifice; and, upon them, a blunt-pointed bistoury, guarded with linen towards the handle, so as to leave no more than about three-quarters of an inch free towards the extremity. This portion being turned upon the edge of the cicatrix, it was carefully divided, as in the manner recommended for the division of a hernial stricture.

The instrument was three times thus introduced, before the section was completed; and, at each step, both Mr. Lever and Mr. Batcheler examined the progress made. Altogether, nearly an inch in length was divided: almost no blood followed, nor did the incision occasion any pain.

This extreme caution in the manner of operating proceeded from two causes: 1st, From the fact that the uterus contracted almost as soon as the finger touched it, and it was hence difficult to insert the instrument: and, 2dly, From an apprehension, which Mr. Lever and myself entertained, that the bladder might have acquired adhesions posteriorly, consequent upon the former operation; for it was plainly felt, by each of us, in very unpleasant proximity to the part about to be incised, and it was, moreover, somewhat prolapsed into the vagina. For a brief space of time, after this, there was a temporary lull in the uterine contractions, and she appeared rather faint: a little brandy and water was given, and the pains soon returned; but little progress was made for about three-quarters of an hour, when, under a severe pain, Mr. Batcheler, who was in anxious attendance, thinks an additional rent took place. The head now immediately left the uterus, and the delivery was completed without further impediment. The child was asphyxiated at the time of birth, and with difficulty restored by Mr. Batcheler. It was a female, and quite full grown."

Some pain over the pubes followed, but it yielded to Dover's powder, or to nature, and, on the 23rd, she could take a walk of four miles. The child died on the 6th.

Mr. Tweedie mentions the following additional facts:—

The child which was born to her in her first confinement is well and thriving: it is rather more than two years old. She suckled it fifteen months, from the right breast alone: the left, which had no nipple, had, after a time, ceased to secrete milk, and was shrunk up. During the time she was suckling, she believes she miscarried twice; once when two months advanced, and again when three months gone. She had no attendance either time, and was not laid up. When not pregnant, she always menstruated every month, even during the suckling:

hence arose the confusion in her calculation this time. She has been generally in good health: she has had, occasionally, a slight watery discharge in the intervals of the catamenial periods, though not nearly to the same extent as was noticed prior to her first gestation. Marital congress has always occasioned pain, especially since the period of her first confinement.

Mr. Tweedie offers the following as the probable explanation of the circumstances of the case.

"It is an ascertained fact, that our patient has no cervix uteri; that is to say, that portion of the uterus commonly called the cervix, which protrudes from its body into the vagina, which is not covered by peritoneum, and which, in the unimpregnated state, constitutes nearly a third part of the length of the whole organ, is in this woman totally wanting.—As a curious coincidence with this malformation, it is worth mentioning, that there is no nipple in the left breast. There being thus no cervix, it is evident that the glandular or follicular structure of the part cannot exist; but it does not therefore follow that there was no opening into the womb prior to impregnation. We believe there was an opening, but not surrounded by the glandular structure which naturally exists here: hence, when impregnation took place, the ordinary mucous secretion could not be found, to seal it up; and is it very unreasonable to imagine, that, under this malformation of parts, adhesive matter, instead of mucus, might have been poured forth, and thus, by adhesion, as pregnancy advanced, the orifice have become entirely obliterated?

With regard to the second confinement, it is stated, that an os uteri existed, of irregular form, posteriorly and laterally appearing almost continuous with the vagina, but anteriorly bounded by a strong, firm, unyielding, rigid edge, upon which one of the cicatrices of the first incision could be plainly felt, passing forwards towards the left ilio-pubic junction. It is probable, that, before we were called to her, the labour-pains had already separated the adhesions; which were described, after the first labour, as proceeding backwards from the artificial os uteri which Dr. Ashwell had cut; and in this way had occasioned the irregular figure which we found at the time of our examination. At all events, this fact tends, in my mind, strongly to corroborate the accuracy of our report two years ago; for it is certain that this was the opening which had been made at the former confinement, because upon its border terminated the scar of adhesion above described. And there was no other opening nor cicatrix to mark where another might have existed from the vagina into the womb."

Mr. Tweedie quotes some other cases, which it does not seem necessary for us to particularise. With the single, but not uncalled for, observation, that his communication is both interesting and instructive, we pass to the next paper.

### VII. On Incision in Cases of Occlusion and Rigidity of the Uterus. By Samuel Ashwell, M.D.

Dr. Ashwell's opinion is justly prized by those who are aware of his zeal and attainments. We cite it, therefore, with pleasure, and our readers may consult it with advantage. Dr. Ashwell observes:—

"I am desirous to make a few brief and practical observations on the safety of incision, in most cases of entire closure of the os uteri; and in some, of the rare examples of its extreme rigidity at the time of labour. It is essential to be thus explicit in defining the cases where such an operation is required, to guard against a rash and unwarranted use of the knife; and it may unhesitatingly be affirmed, that the practitioner, before such a procedure is determined on, ought to be most fully convinced that the patient's safety can be better secured by this than by any other method. It may too be observed, that the medical attendant should not, except when a consultation cannot be obtained, adopt the plan now proposed on his own responsibility.

He thinks it may be shewn:—

1st, That incision is the safest remedy, where the os is in a state of firm and complete closure; or, in other words, where the uterus, so far as its lower orifice is concerned, is imperforate: and,

2ndly, That in examples of such extreme rigidity of the os, where, after hours of strong uterine effort, the power of dilatation is entirely absent, whether such rigidity arise from disease in the structural organization of the part, or has resulted from previous laceration and ulceration, incision is the best and safest treatment; far preferable to protracted and powerful dilatation of the os by the finger; or, on the principle of non-interference, to leaving the case to the natural efforts.

*a.* Dr. Ashwell alludes, without approbation, to the opinion that, in many instances, an oblique cervix, the os being situated unnaturally high, is mistaken for occluded os uteri.

He goes on to state:—

There can be but little difficulty in the diagnosis of instances of complete and firm closure of the os. When parturient effort is really established, the lower portion of the uterus, in the form of a tense and large globular mass, is generally forced down very low, sometimes so far, as nearly to reach the external entrance of the vagina. Thus a finger—at all practised in these inquiries—must detect an aperture, if there be one; and, if not, the spot where the os uteri, at the time of conception, had been.

A repetition of uterine action will afford abundant opportunities for careful re-examination; so that no apology for indiscreet and dangerous delay can exist. If, too, a spot shall be discovered—more depressed, and of different structure to the surrounding parts, indicating the site of the os uteri at the time of impregnation, it is impossible then to doubt about the nature of the case.

The treatment of such a case naturally claims attention. He intreats practitioners to pause before they determine on large bleedings and delay—both dangerous. Two other remedies offer themselves:—

1. Such an amount of pressure, by the finger, female catheter, sound, or bougie, as shall puncture or open the occlusion: and,

2. Incision with a bistoury or knife.

"When the occlusion," he goes on to remark, "is slight, depending on a thin membrane, interposed between the margins or filling up the circumference of the os, similar to the membrane found between the adherent labia of female children, the finger, as recommended by Nægele, in his very interesting Thesis, may produce a separation or orifice; or, if this digital pressure be insufficient, the catheter, sound, or bougie, may enable us to do what we wish. It is afterwards to be expected, if the structure of the cervix be healthy, that dilatation of the os will proceed as satisfactorily as in the many cases where this orifice is naturally small. In such, we rarely find the power of dilatation absent."

This method of proceedure is, he conceives, inapplicable, where the interposed cellular membrane, shutting up the os, has become thoroughly organized and firm; so much so, indeed, as effectually to have resisted twelve, twenty, or thirty hours of most urgent uterine effort. The forcible use of the finger or catheter must necessarily give rise to contusion, and this would be not unlikely to give birth to partial, if not general uterine inflammation. It may too, perhaps, he adds, be fairly assumed, that the risk of unlimited laceration of the uterus and adjacent parts is much less, where incisions of tolerable extent have been discreetly made, than where merely a diminutive central aperture has been formed by a blunt instrument.

*b.* Having proved, for we conceive it *is* proved, the value of incision in cases of occlusion of the cervix uteri, Dr. Ashwell next attempts to prove that incision may also be practised with advantage in cases where the os uteri is excessively rigid.

"It will not be supposed that I recommend the knife to be at once employed in these more complicated maladies; but I am confident—so far, at least, as it is possible to be confident, in cases where a high probability must be our only guide—that where fatal results have occurred, they might often have been prevented by timely incision of the parts. But it has too often happened, as the appended cases shew, either that the operation has been performed too late, or that a too powerful dilatation by the finger, and an unwise reliance on the natural efforts, have altogether superseded its employment. Examples of entire occlusion without disease, like those to which I have already alluded, are much more rare than extreme rigidity of the cervix and a diminished os: nor will it be found quite so easy in the latter, as in the former class of cases, to determine the precise moment when bleeding, diaphoretics, fomentation, and delay, are to yield to the use of the bistoury; still the general safety of incision, and the known and imminent danger of protracted and severe uterine effort and contusion, ought to induce an *earlier*, rather than a *deferred* operation. A careful perusal of the cases and authorities appended to this essay, especially Smellie's can scarcely fail to impress this conviction. In every instance, or nearly so, where the division of the morbid structure has been made prior to the occurrence of inflammation and sinking, it has succeeded; and, generally, with the fewest possible bad symptoms. Where, on the contrary, violent uterine action, contrary to the sagacious directions of the experienced Dr. Hamilton, has been allowed to go on for a great number of hours—say, twelve, fifteen, twenty-four, or even a longer period—the result has been generally unfavourable, often fatal; and still more certainly so, where, during a portion of this time, powerful dilatation has been long and forcibly employed. Dilatation by the fingers is not the same operation here, as to its safety, which it is found to be in examples of rigidity not dependent on, or associated with, local or structural malady. It is true, that, in transverse and placental presentations, artificial dilatation is often practised with safety and advantage. Neither the mouth nor neck of the womb, both being healthy, suffer from the process: prevention of hæmorrhage, and a freedom from useless and exhausting pain, are the results of the process: but where the cervix is rigid, contracted, and diseased, and the os so small as scarcely to be recognised, powerful and long-continued artificial dilatation *must be a dangerous remedy*. It is scarcely to be expected that it should relax the parts and lead to dilatation: it is much more likely that it should irritate, and thus induce inflammation, gangrene, and death."

Dr. Ashwell goes on to observe:—The simplest, perhaps, of the examples of rigid os uteri is where a very contracted orifice is surrounded by a structure almost entirely undilatable. In such a case, although there may be little if any indication of organic change, still, if there be a total absence of the power of dilatation, after the use of free venesection and antimony—time having been allowed for their beneficial effects—such a case cannot be long trusted with safety, either to the natural efforts or artificial dilatation. Other examples are not so simple as this. Many, probably the majority, are the consequence of some previous morbid occurrence. The os and cervix may have been injured in a former labour: abscesses, ulcerated surfaces, and cicatrizations, may have taken place: thus the uterine orifice may have become nearly, if not entirely closed; and the relative situation of the urethra, bladder, and vagina, so altered, as to render the division of parts much more difficult and hazardous: or it may be, that a hard tumor, or a more malignant and active deposit, has imbedded itself in these parts, totally altering the os and the natural structure of the cervix. In one essential particular, all these varieties will be found to agree; viz. in the difficulty with which the os and cervix are dilated; while in some, and not a few of them, the susceptibility of dilatation will have been entirely destroyed.

Supposing the incapability of dilatation satisfactorily established, supposing that bleeding and every adjuvant remedy have been fairly, but unsuccessfully tried, Dr. A. believes that we ought not to hesitate about incising the cervix, where the violence and frequent return of the uterine effort threatens rupture of the womb. If there be distressing and constant pain about the neck or body of the uterus, or in any other part; if the countenance becomes turgid and dark; if perspiration issues at every pore, and the pulse is full, strong, quick, and incompressible: and if these symptoms continue, although perhaps somewhat lessened by bleeding and antimony; there can be no doubt that recourse should be had to the incision. It is impossible to fix a precise limit during which a patient may be safely left to her own unaided efforts: time cannot be the sole ingredient, although an essential part of every rule, regulating interference in obstetric cases.

"The operation," he remarks, "in any of the cases, whether it be on an os firmly closed, yet without organic change—or on an os very diminutive and contracted, with or without surrounding disease, but entirely undilatable—is, generally, easily performed. A probe-pointed knife or bistoury is the instrument most safely used;—the woman lying, either on her left side or on her back close to the edge of the bed. The forefinger of the left hand is to be carried to that spot of the cervix intended to be cut: afterwards, the knife or bistoury is to be cautiously conveyed, along the finger in the vagina, to the spot already mentioned; and if its point be gently pushed against the uterine structure, it will completely incise the parietes. In Mrs. Purcell's case, I carried the knife, first of all, forwards, toward the neck of the bladder (which was empty), carefully avoiding it; afterwards towards the sacrum, making an incision about two inches long. The liquor amnii will necessarily escape as soon as the first incision is made. The instrument may now be carefully withdrawn, and the further dilatation left to nature. It is scarcely to be expected that all rending should be avoided; but the extent of the tearing is, as has been already stated, generally confined within the limits of the vagina. I have no experience of the better effect of a crucial incision, in preventing extensive laceration; but I am favourably inclined to it. It is not probable that much blood will be lost during or after the operation: in my own cases, only a few drachms escaped. If there should be fainting and collapse, after the incision of the parts, brandy and ammonia may be freely exhibited. It is a necessary preliminary step that the bladder and rectum be emptied of their contents. In Mrs. Purcell's case, the birth of the child was accomplished, in both instances, without instrumental aid; but the forceps is not unfrequently necessary safely to terminate the labour."

### VIII. OBSERVATIONS ON ABDOMINAL TUMORS AND INTUMESCENCE: ILLUSTRATED BY CASES OF RENAL DISEASE. By R. BRIGHT, M.D. F.R.S. &c.

The present is a sequel to those valuable papers on Abdominal Tumors, of which we have, in former numbers presented an account. Of Dr. Bright's unwearied diligence and untiring zeal it is not necessary for us to speak.

The object of this communication is to exhibit such abdominal tumors as depend on enlargement of the kidney. The affections of that organ unattended with augmentation of dimensions do not come within its scope.

The chief diseases which have given rise to renal tumor in Dr. Bright's experience have been—when numerous cysts have been developed in the substance of the kidney; when puriform matter has collected in the pelvis, and converted the distended kidney into a bag of pus;—when fungoid or malignant changes have taken place in the kidney;—when fungoid matter or blood has been accumulated in the pelvis. Dr. B. has known the enlarged kidney to be mistaken for disease of the spleen—of the ovary—of the uterus—and for a tumor developed in the concave part of the liver: nor is it perhaps possible, by the greatest care and the most precise knowledge altogether to avoid such errors.

Of the anatomical position and relations of the kidneys it does not seem requisite for us to speak. After skimming along them, Dr. Bright concludes :—

"Although closely attached to the muscles of the loins in its natural condition, yet, in those diseases in which it most rapidly increases, the enlargement shews itself much more towards the anterior part of the abdomen than towards the loins; not only because the firm structure of this part is more calculated to conceal a tumor, but also because in the other direction it meets with less immediate resistance; so that it often happens, while we are examining the lumbar region with the greatest care, and obtaining but a doubtful evidence of fulness and hardness by the eye, and by the touch, and by careful comparison of the two sides, we can scarcely place the hand upon the anterior or even the lateral part without becoming at once sensible of the existence of a distinct tumor;—and then, probably, by pressing that tumor backward, the other hand clearly informs us of its connection with the loins. The part in which the tumor is felt, will, of course, vary according to the nature of the disease, and to the portion of the kidney which it occupies; and in some cases, where the whole substance of the organ is so diseased as to contribute pretty equally to the enlargement from the beginning, the hardness or tumor will be early detected in the loins. Thus we find, that a rapidly-increasing fungoid disease in the right kidney may be chiefly perceived pushing its way beneath the liver; a large collection of pus, or other accumulation, enlarging the natural cavity of the kidney, will probably be felt most distinctly towards the anterior part, and, from the assistance of gravitation, will occupy a place between the umbilicus and the crista ilii; while, on the contrary, a kidney enlarged by numerous cysts, affording a comparatively solid and uniform increase to the whole organ, will be most distinctly felt occupying the lumbar space, and giving solidity and firmness to that part. It will likewise be found that when inflammation has pervaded the kidney, or attacked the external part, it will be bound down to its natural situation, and completely fixed in the loins; not advancing, as the fungoid kidney often does, towards the anterior part of the abdomen."

*a*. Enlargement of the right kidney may be mistaken, if it makes its way forward, for an enlargement of the liver, for pyloric disease, for a glandular disease about Glisson's capsule, for disease of the colon or cæcum, or for enlargement of the ovary or uterus.

To distinguish it from enlargement of the liver, Dr. Bright judiciously advises us to attend to its relations to the ribs. If the liver, he remarks, be healthy, we shall probably find that the tumor, as the patient lies on his back, instead of passing fairly under the ribs, dips downwards, so as to allow the finger to lie between them and the upper part of the tumor. Again, we seldom have disease of the liver to the extent which is here supposed, without producing some pretty decided symptoms, either in the colour of the eye, or the tinge of the skin, or in the deep colour of the urine, or the diseased secretions evinced by the stools.

Disease of the liver may be combined with that of the kidney, as in malignant disease. It seems to us that, under such circumstances, a precise diagnosis is a matter of very little consequence. But Dr. Bright hopes that even here it is possible, that should the urine be altered in its character, more particularly if it contain pus, and, in addition to other symptoms of hepatic affection, should the peculiar hard tubera, which, under such circumstances, often form in the liver, be perceptible under the ribs, we might come to a correct diagnosis as to both the diseases. We may, moreover, in this case, derive much assistance, from ascertaining by the feel and by percussion, the exact situation of the hollow intestines, for although it is true that they suffer great displacement, yet, if we find any of them anterior to the tumor, and lying over it, we may generally infer that the tumor does not form a part of the liver; as it is very improbable that such a growth should arise from the concave surface of the liver, as to have any portion of the intestine in that situation.

The cæcum and ascending colon are liable to disease, and to enlargement both from the presence of flatus and from accumulation of fæces, and occasionally from other accumulations. In all these cases the disturbance of the functions of the large intestines will furnish useful assistance to our diagnosis. We shall, in cases of flatulent distention, ascertain the fact by percussion, and by the rapid alterations which the tumor undergoes. In cases of fæcal abscess, a disease very common from lodgments taking place in the vermiform process, the febrile symptoms generally run much higher than in renal tumors: there is often external inflammation, and much tenderness; and, above all, the tumor is often found too low in the iliac region to be probably produced by the kidney, though Dr. Bright has known such abscesses discharge their contents almost in the lumbar region. In cases where concretions have formed, occupying a large portion of the cæcum, considerable difficulty may arise in the diagnosis, if we simply look to the tumor; but the disturbance of the bowels, the intense abdominal pains, and tormenting collection of flatus, will be our guide.

Perhaps, says Dr. Bright, the most frequent mistake is to consider the enlarged kidney, an ovarian or uterine tumor. The history of the disease offered by patients is fallacious. Dr. Bright contributes some hints towards their discrimination.

"The present situation of the tumor will enable us to discover that it is not connected with the pelvic viscera; and usually there is a distinct sulcus into which the hand may be placed, between the tumor and the pelvis. Another point to be attended to, is, the situation of the hollow viscera; which, by careful examination, will be found to overlap, or to pass over the surface of the tumor; —and this, together with the history of its growth, ought sufficiently to direct our judgment. Occasionally, the ovarian tumor assumes such varieties of form as to deceive the most experienced: and an instance very lately came to my knowledge, when several, who were consulted, altogether denied the ovarian origin of the tumor, and ascribed it to the liver; though, after death, it turned out to be ovarian. In this instance, the absence of any hollow viscera anterior to the tumor would have prevented the supposition of its being kidney at least; though the same might not hold good as to the liver. When the kidney has descended almost to the pelvis, and approached the middle line of the abdomen, it has been mistaken for uterine tumor; but an examination of the neck of the uterus, and of the uterus itself, in the usual way, will come in aid of the indications of which I have been speaking, as applicable to the ovary."

*b*. Enlargement of the left kidney may be mistaken for the spleen, for the descending colon, for the ovary, and for the uterus.

"The enlarged spleen is situated more anteriorly; and, in its descent, though occasionally much rounded, generally presents a more-defined edge than the kidney, often suffering the fingers to be introduced beneath it; and it is sometimes notched at the edge: it has none of the hollow bowels to interrupt the uniform surface of the tumor. The spleen is sometimes inseparably attached to the tumor of the kidney, of which we have a specimen in Guy's Museum: but when this is the case, the spleen generally occupies the posterior part of the left hypochondrium, and therefore adds little either to the facility or the difficulty of diagnosis. Nearly the same remarks are applicable as regards the descending colon and its accumulations, and also the left ovary and the uterus, as have already been made, when speaking of the right."

*c*. There are three forms of tumor connected with the kidney, the possibility of the occurrence of which should be remembered. The one is real acephalocyst hydatid, which may develop itself in the kidney, or be attached to it. Dr. Bright has not actually met with this. The next is tumor from disease of the renal capsule; this organ being liable to both scrofulous enlargement, which our author lately saw, and to malignant tubera, which he has seen several times. The third form of tumor, arising in connection with the kidney, is, the simple distention of the pelvis and ureter, with the natural secretion, owing to obstruction in the ureter and bladder.

When hydatids, says Dr. B. in his suggestions for a diagnosis, are developed in the kidney, they can only be ascertained by their situation, and by the means applicable to the detection of hydatids generally. The tumor of the renal capsule must at present, from its situation as a portion of the kidney itself, and from our great ignorance of the function it performs in health, be almost beyond the scope of decided diagnosis, but may be suspected from the situation of the tumor in the upper part of the kidney; but the liver lying before it, and the muscles of the back and ribs behind it, it is very improbable that it would be detected. In the case of scrofulous disease, which he witnessed, the tumor, of the size of a small egg, was fixed to the upper and posterior part of the liver, in which it was almost imbedded. The dilated ureter may be detected by its situation, which may, if not close to the kidney, be sufficiently characteristic, and by its elastic feel; —and it will be certainly detected, if its contents can be evacuated into the bladder.

d. "Supposing that our diagnosis has been satisfactorily formed, and that a tumor of the kidney has been discovered, it still becomes desirable, if it be possible, to establish the exact nature of the disease to which the increased bulk of the organ is to be ascribed;—a problem, which is even more difficult to solve than the former. We have often to look back into a long history; and there is every reason to believe that, in many cases, there is a successive or simultaneous development of different diseases; so that it is possible to come to a right conclusion as to part of the disease, and yet not discover the whole. Thus we shall find, by comparing histories with post-mortem appearances, that in one case almost undoubtedly a calculus has been deposited in the pelvis of the kidney, has excited suppuration, and a tumor has been formed; but that, after it has existed some time, malignant action has been set up. In other cases, we shall find that an injury to the loins has been followed by hæmaturia, and that, after a time, a malignant disease has established itself. Again, we shall find a calculus to begin, and this followed by a collection of pus in the pelvis, and this succeeded by a granular change in that portion of the renal substance which has not undergone absorption;—or we shall have reason to believe that the granulation of the kidney has taken place; and that afterwards, or at the same time, cysts have been formed in the cortical substance, and a tumor of the kidney has been the consequence;—and, as the post-mortem appearance, in conjunction with the history, is capable of bringing us to such conclusions, so the history by itself, in conjunction with the physical or local, and general symptoms, may bring us nearly to the same point:—and I will now proceed to refer to some of the data which may serve to guide us in the inquiry."

e. The two symptoms, most remotely connected, in the majority of cases, with diseased kidney, are hæmaturia and the passing of small calculi by the urethra. Neither necessarily indicates or leads to organic alteration, but both, when the change is discovered, throw some light on particular cases.

It is certain, continues Dr. Bright, that hæmaturia takes place under a variety of circumstances: some states of congestion and inflammation, such as often occur in consequence of intemperance, or after exposure to cold, or subsequent to scarlatina, will produce hæmaturia; and this will probably never be followed by enlargement of the kidney, or, at all events, never to the extent of producing sensible tumor; and of this we may have almost hourly experience. A general hæmorrhagic tendency of the system will often shew itself by hæmaturia; in which case, under particular circumstances, extensive ecchymosis will be produced in the pelvis of the kidney, but may subside without causing any tumor of that organ. The more local causes of hæmorrhage, as obstruction to the circulation through the heart or even the large viscera of the abdomen, may produce slight hæmaturia, without any enlargement of the kidney following; but where a profuse hæmorrhage takes place, or a tendency to it shews itself, such mischief frequently follows as leads to tumor of the organ. This hæmorrhage is probably not to be considered so much the result of any one form of disease tending to enlargement, as the source from which irritation is set up. The coagulum forming, and not capable of immediate expulsion, produces irritation, and assists the deposit or the accumulation of calculus; or, by retaining the urine in the pelvis, produces inflammation and suppuration there. Every instance of hæmorrhage which can be fairly traced to the kidney, and in which the entire blood comes away in a form capable of coagulation, must be looked upon with fear, as likely to lay the foundation for some organic change. What that consequence may be depends rather on the tendency of the system or the part, than on the hæmorrhage per se.

When hæmorrhage occurs in the more-advanced state of the disease, I should look to the circumstance of its being pure, or mingled with pus, as important in a diagnostic point of view; when it is pure, forming clots which are perhaps moulded to the shape of the passages, if I found a tumor, I should consider it probable that the kidney was pervaded by cysts, or that, in some way, great obstruction was experienced to the passage of the blood through the remaining substance of the kidney; still the diagnosis would be modified by the character of the tumor: if it were hard, resisting, and chiefly lumbar, I should be more confirmed in this belief; and if, in addition to this, I found that the urine, when perfectly clear of blood, after the hæmorrhage had for some days completely subsided, was still albuminous, I should very confidently expect some such degeneration in the substance of the kidney as I have described, intermixed possibly with granular deposit.

When hæmorrhage occurs in smaller quantity, but mingled with pus, and generally subsiding rather more slowly than the pus, so as to form a fringe-like deposit on its surface, it probably bespeaks some local bleeding from the pelvis, either depending on the presence of a rough calculus, to a small extent lacerating or rubbing the membrane, or more commonly depending on a tendency to fungoid growths beginning to arise from it."

If small calculi, our author goes on to remark, have been passed in the early part of the history of a renal tumor, the natural conclusion to which we come is, that some similar formation having taken place within the pelvis of the kidney, and having been unable to find its way down the ureter, the pelvis has been irritated either by the calculus or much more likely by the retained urine, and pus has accumulated in the cavity and distended it;—but this seldom happens, without pus being actually passed.

*f*. When a large tumor is formed by the kidney, and neither calculus nor blood nor pus has marked the progress of the disease, Dr. Bright would feel inclined to consider this a fungoid or malignant disease.

*g*. Circumstances connected with the character and growth of the tumor, independently of the nature of the renal discharge, give some hints on its nature.

If the tumor be hard and insensible, and lodged in the lumbar region, we should incline to the supposition that it was neither enlarged from pus nor from fungoid growth, and may probably be changed in structure throughout, or pervaded with cysts.

If the tumor appear to have increased very quickly, and especially to have grown irregularly, projecting in particular parts, advancing upon its upper part towards the scrobiculus cordis, rather than descending towards the pelvis, or increasing regularly towards the mesial line, we conclude that the disease is rather a fungoid or malignant growth, than the product of simple inflammation.

If the tumor have enlarged regularly, or with only certain moderate elevations, forming a somewhat ovoid body, or have become soft or fluctuating in parts, then, even if pus had not been ascertained in the urine, we should be inclined to ascribe the tumor to a collection of pus.

“When speaking of the diagnosis of these tumors, it is impossible to pass over in silence the importance of the very valuable test of pus which was first pointed out by Dr. Babington, in a former Part of these Reports. It occasionally happens, that very large deposits, both of the lithates and of the phosphates, are thrown down from the urine; which, on first being seen as they have formed or subsided in the vessel, bear so much the appearance of pus, that not only the patient, but the practitioner who has not paid a good deal of attention to the subject, has been deceived. The lithates are at once detected by their entire disappearance, if heat be gently applied, or a few ounces of warm water be added to the urine; and, indeed, we may generally learn, by inquiry from the patient, that the urine was perfectly clear when first passed, becoming turbid only as it cooled. The phosphates, however, are of less easy detection; but if there be any suspicion that the deposit is purulent, by pouring off the clear fluid, and adding to the deposit a few drops of the liquor potassæ or the liquor ammoniæ, and agitating them together, we find that, if it be pus, it is converted, in the space of a few seconds, into a substance resembling the most tenacious mucus: and this process is often carried on previously to the discharge of the urine; for if the urine become alkaline in the bladder, as it often does in paralysis and in some other cases, this conversion of pus into mucus takes place in the bladder itself;—and this has probably often misled the practitioner, who has been in the habit of regarding the large quantity of ropy mucus, which is sometimes found at the bottom of the vessel, as a secretion of the mucous membrane of the bladder, whereas it is, in reality, very often only a puriform secretion of the kidney, which has undergone conversion in the bladder. I have, at this time, under my care a lady labouring under copious purulent discharge from the kidney, which, on one or two occasions, has been passed in the form of mucus, owing to the administration of alkaline remedies.”

Dr. Bright winds up these observations, and ushers in his cases with these general remarks:—

“The history of those diseases which induce tumors of the kidney will, of course, vary greatly, according as they depend on simple inflammation, on a scrofulous constitution, or as they are more or less malignant in their character. The approach is often slow and insidious; and when the tumor has shewn itself, or by other indications the established disease is discovered, the patient is often able to refer back to some period when unusual exposure to cold, or some sudden jerk, or some accident to the loins, may here be the presumed exciting cause: very frequently we find in females, that although some other cause may be discovered, and perhaps some symptoms may have previously occurred, yet the malady had never shewn itself decidedly till after pregnancy, and the tumor has first been discovered as the patient recovered from her confinement: and where this is the case, it is reasonable to suppose that the pressure of the uterus, having obstructed the passage of urine along the ureters, may have acted as an aggravating, or perhaps as an exciting cause of suppuration or of malignant disease. There are, however, some other circumstances connected with pregnancy, which act, in the first place, as throwing a difficulty for several months over the detection of a tumor in the abdomen, and then calling the attention more directly to its existence. In some cases we can distinctly trace that the obstruction and irritation resulting from stricture, from disease of the bladder, or from stone in the bladder, or kidney, have been the exciting causes of the disease.—Diseases of the kidney, tending to the formation of tumor, are confined to neither age nor sex. Scrofulous disease with enlargement, and fungoid diseases of the most remarkable and rapid growth, occur in children of the most tender age: indeed the kidneys of children are very susceptible of disease, both functional and organic. In more-advanced age, the obstructions in the urinary passages increase, and formidable calculous diseases multiply."

The cases themselves are twelve in number. The first is one of tumor of the kidney, from numerous cysts found in its substance—the second, suppuration of the kidney, from stricture of the urethra, attended with perceptible tumor—third, tumor formed by the kidney—the pelvis being distended with pus—fourth, tumor from puriform collection in the kidney, first perceived after parturition, but apparently depending on the presence of a calculus—fifth, large tumor formed by the left kidney, supposed to be uterine; the pelvis being distended with grumous matter, and the substance of the organ suffering, together with the liver, from malignant disease—sixth, tumor formed by the kidney, dilated with puriform fluid—seventh, tumor of the kidney, with copious puriform discharge through the urethra, and probably through the bowels—eighth, tumor formed by the left kidney, discharging pus copiously both by the urethra and the rectum, depending on a large renal calculus—ninth, cerebriform tumor of the right kidney; supposed to be a tumor arising from the concave surface of the liver—tenth, tumor of the kidney from fungoid disease mistaken for the spleen.—Death by rupture into the peritoneal cavity—eleventh, fungoid tumor of the kidney, affording the appearance of two tumors—twelfth, fungous disease of the glands of the mesentery, resembling enlarged kidney.

We must refer the curious to Dr. Bright's paper, should they wish to learn the particulars of these cases. Appended to them are the novissima verba of the author. He thinks they prove, what indeed was but too certain before their publication, the extreme difficulty of diagnosis. That difficulty partially, if not mainly, hangs on the circumstance that there are two kidneys, and that one takes on itself much of the function, snatched by disease, from the other. The healthy kidney, it is well known, often becomes hypertrophied, a proof and a guage of its increase of action.

Dr. Bright makes some final remarks on suppuration of the kidney, with which we shall close our notice of his paper.

"In the foregoing cases we have instances of suppuration of the kidney of two kinds—where the disease seems to have begun in the substance of the organ itself; and where it has been, apparently, almost entirely a purulent secretion from the pelvis;—and this latter is by far the most common in cases which afford any enlargement of the organ capable of being discovered before death. In these cases, the whole kidney becomes reduced almost to the state of a thick sacculated membranous bag; the lining of the pelvis being brought so nearly in contact with the external tunic, that nothing but a thin and condensed layer of the substance of the kidney separates them: but there is still no apparent breach of continuity or suppuration in the substance of the organ, nor does it appear to have commenced in that way. Frequently, however, after some time, fungoid growths spring from the lining membrane; and frequently the tendency to extend and to suppurate is not bounded by the organ itself; and the most common result is, that an opening is formed into that portion of the colon which passes over it. This process, even before the communication is fully formed, is often attended with diarrhœa; which, in the already weakened condition of the patient, adds greatly to the urgency of the disease; and when the ulceration has extended into the intestines, much puriform matter is evacuated. At other times, the tendency seems to be rather to the formation of an external opening. I do not remember to have met with a case in which it has opened of its own accord in that way; but where the fluid has approached so near the surface as to lead to the evacuation by the lancet or the trochar, it has again and again accumulated. There is at least a third way in which the pus may escape; and that is, by ulceration or rupture into the cavity of the abdomen. It is not very probable that this effusion of pus should take place by ulceration, because it very generally happens that the adhesive process prevents such a result; but it is more probable by accidental rupture, and then would most likely prove fatal."

It is unnecessary to say more than that the present is characterised by the same minuteness and accuracy, which our author's former contributions to pathology have displayed.

---

### MASSACHUSETTS' GENERAL HOSPITAL.

